# Complete genome sequence of *Mesorhizobium opportunistum* type strain WSM2075^T^

**DOI:** 10.4056/sigs.4538264

**Published:** 2013-12-15

**Authors:** Wayne Reeve, Kemanthi Nandasena, Ron Yates, Ravi Tiwari, Graham O’Hara, Mohamed Ninawi, Olga Chertkov, Lynne Goodwin, David Bruce, Chris Detter, Roxanne Tapia, Shunseng Han, Tanja Woyke, Sam Pitluck, Matt Nolan, Miriam Land, Alex Copeland, Konstantinos Liolios, Amrita Pati, Konstantinos Mavromatis, Victor Markowitz, Nikos Kyrpides, Natalia Ivanova, Lynne Goodwin, Uma Meenakshi, John Howieson

**Affiliations:** 1 Centre for Rhizobium Studies, Murdoch University, Western Australia, Australia; 2 Los Alamos National Laboratory, Bioscience Division, Los Alamos, New Mexico, USA; 3 DOE Joint Genome Institute, Walnut Creek, California, USA; 4 Oak Ridge National Laboratory, Oak Ridge, Tennessee, USA; 5 Department of Agriculture and Food, Western Australia, Australia; 6 Biological Data Management and Technology Center, Lawrence Berkeley National Laboratory, Berkeley, California, USA

**Keywords:** root-nodule bacteria, nitrogen fixation, evolution, lateral gene transfer, integrative and conjugative elements, symbiosis, *Alphaproteobacteria*

## Abstract

*Mesorhizobium opportunistum* strain WSM2075^T^ was isolated in Western Australia in 2000 from root nodules of the pasture legume *Biserrula pelecinus* that had been inoculated with *M. ciceri* bv. biserrulae WSM1271. WSM2075^T^ is an aerobic, motile, Gram negative, non-spore-forming rod that has gained the ability to nodulate *B. pelecinus* but is completely ineffective in N_2_ fixation with this host. This report reveals that the genome of *M. opportunistum* strain WSM2075^T^ contains a chromosome of size 6,884,444 bp, encoding 6,685 protein-coding genes and 62 RNA-only encoding genes. The genome contains no plasmids, but does harbor a 455.7 kb genomic island from *Mesorhizobium ciceri* bv. biserrulae WSM1271 that has been integrated into a phenylalanine-tRNA gene.

## Introduction

*Biserrula pelecinus* L. is an autogamous annual legume species that is common, though never dominant, on coarse textured and acidic Mediterranean soils [1] and can often be found with other annual legumes including subterranean clover (*Trifolium subterraneum*) and serradella (*Ornithopus*) [2]. This reseeding legume was introduced to Western Australia in 1993 in a pasture legume breeding and selection program that sought to develop new pasture legume options for the sandy surfaced duplex, acidic soils in Western Australia, to improve soil fertility and farming system flexibility [1]. At the time of introduction, the Australian resident rhizobial populations were not capable of nodulating *B. pelecinus* [1,3] and a Mediterranean strain *Mesorhizobium ciceri* bv. biserrulae WSM1271 had to be used as an inoculant to establish an effective nitrogen fixing symbiosis. After 6 years of cultivation of *B. pelecinus* under field conditions, an isolate (designated WSM2075) was recovered from root nodules of plants grown near Northam, Western Australia that displayed an ineffective symbiotic phenotype [4]. Accumulated evidence revealed that WSM2075 had gained the ability to nodulate (but not fix with) *B. pelecinus* by acquiring symbiotic genes from the original inoculant strain *Mesorhizobium ciceri* bv. biserrulae WSM1271 following a lateral gene transfer event [5]. Strain WSM2075 has now been designated as strain WSM2075^T^ (= LMG 24607 = HAMBI 3007) and is the type strain for a new species described as *Mesorhizobium opportunistum* [6]. The species name op.por.tu.nis’tum. L. neut. adj. opportunistum reflects the opportunistic behavior of the organism to nodulate a range of legume hosts by acquiring symbiotic genes [4,5]. *M. opportunistum* WSM2075^T^ is competitive for nodulation of *B. pelecinus* but cannot fix nitrogen [4] and the finding of such strains that have rapidly evolved in the soil presents a threat to the successful establishment of this valuable pasture species in Australia [5].

Here we present a summary classification and a set of general features for *M. opportunistum* strain WSM2075^T^ together with the description of the complete genome sequence and annotation. Here we reveal that a 455.7 kb genomic island from the inoculant *Mesorhizobium ciceri* bv. biserrulae WSM1271 has been horizontally transferred into *M. opportunistum* strain WSM2075^T^ and integrated into the phenylalanine-tRNA gene.

## Classification and general features

*M. opportunistum* strain WSM2075^T^ is a motile, Gram-negative, non-spore-forming rod (Figure 1A and Figure 1B in the order *Rhizobiales* of the class *Alphaproteobacteria*. They are moderately fast growing, forming 2-4 mm diameter colonies within 3-4 days and have a mean generation time of 4-6 h when grown in half Lupin Agar (½LA) broth [7] at 28°C. Colonies on ½LA are white-opaque, slightly domed, moderately mucoid with smooth margins (Figure 1C).

**Figure 1A:**
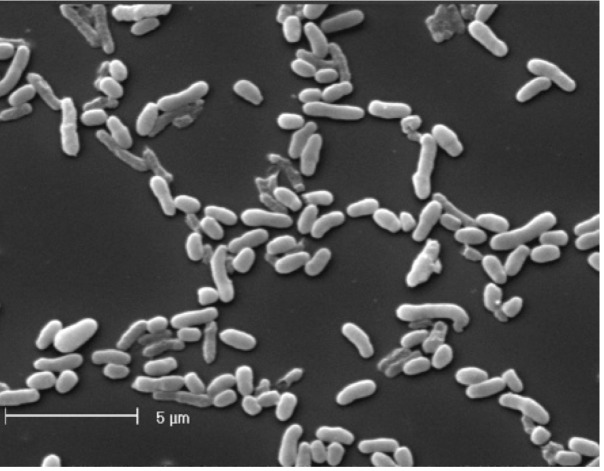
Image of *Mesorhizobium opportunistum* strain WSM2075^T^ using scanning electron microscopy

**Figure 1B:**
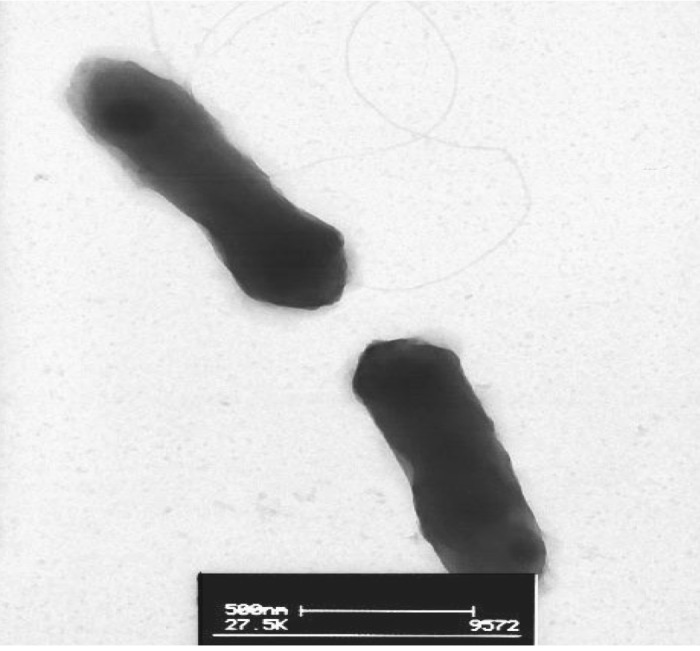
Image of *Mesorhizobium opportunistum* strain WSM2075^T^ using transmission electron microscopy

**Figure 1C:**
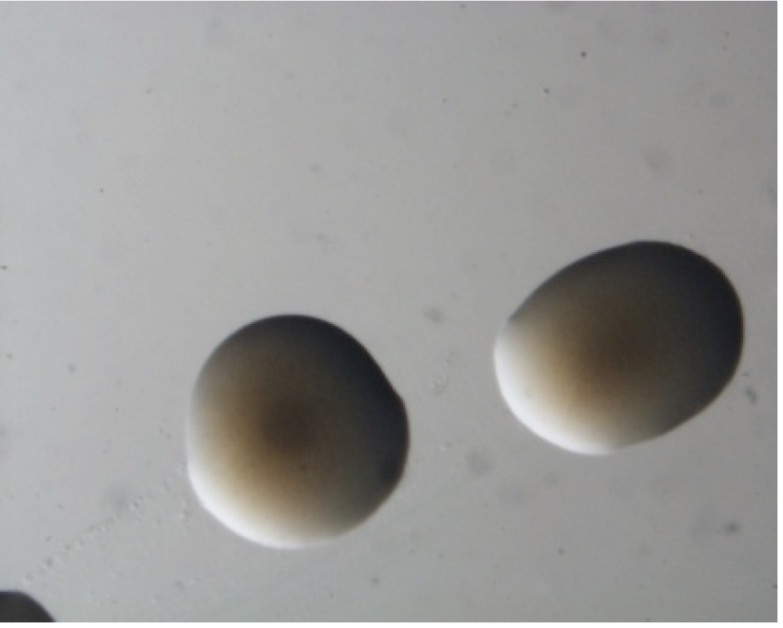
Image of *Mesorhizobium opportunistum* strain WSM2075^T^ colony morphology on a solid medium (C).

Strains of this organism are able to tolerate a pH range between 5.5 and 9.0. Carbon source utilization and fatty acid profiles have been described previously [6]. Minimum Information about the Genome Sequence (MIGS) is provided in Table 1.

**Table 1:** Classification and general features of *Mesorhizobium opportunistum* strain WSM2075^T^ according to the MIGS recommendations [8,9].

**MIGS ID**	**Property**	**Term**	**Evidence code**
	Current classification	Domain *Bacteria*	TAS [9]
		Phylum *Proteobacteria*	TAS [10]
		Class *Alphaproteobacteria*	TAS [11,12]
		Order *Rhizobiales*	TAS [12,13]
		Family *Phyllobacteriaceae*	TAS [12,14]
		Genus *Mesorhizobium*	TAS [15]
		Species *Mesorhizobium opportunistum*	TAS [6]
	
	Gram stain	Negative	TAS [6]
	Cell shape	Rod	TAS [6]
	Motility	Motile	TAS [6]
	Sporulation	Non-sporulating	TAS [16]
	Temperature range	Mesophile	TAS [16]
	Optimum temperature	28°C	TAS [6]
	Salinity	Unknown	NAS
MIGS-22	Oxygen requirement	Aerobic	TAS [16]
	Carbon source	Arabinose, β-gentibiose, glucose, mannitol & melibiose	TAS [6]
	Energy source	Chemoorganotroph	TAS [16]
MIGS-6	Habitat	Soil, root nodule, host	TAS [6]
MIGS-15	Biotic relationship	Free living, Symbiotic	TAS [6]
MIGS-14	Pathogenicity	None	NAS
	Biosafety level	1	TAS [17]
	Isolation	Root nodule of *Biserrula pelecinus L.*	TAS [6,18]
MIGS-4	Geographic location	Northam, Western Australia	TAS [6,18]
MIGS-5	Nodule collection date	August 2000	TAS [4]
MIGS-4.1	Longitude	116.947875	TAS [4]
MIGS-4.2	Latitude	-31.530408	TAS [4]
MIGS-4.3	Depth	10 cm	NAS
MIGS-4.4	Altitude	160 m	NAS

Evidence codes - TAS: Traceable Author Statement (i.e., a direct report exists in the literature); NAS: Non-traceable Author Statement (i.e., not directly observed for the living, isolated sample, but based on a generally accepted property for the species, or anecdotal evidence). These evidence codes are from the Gene Ontology project [19].

**Figure 2:**
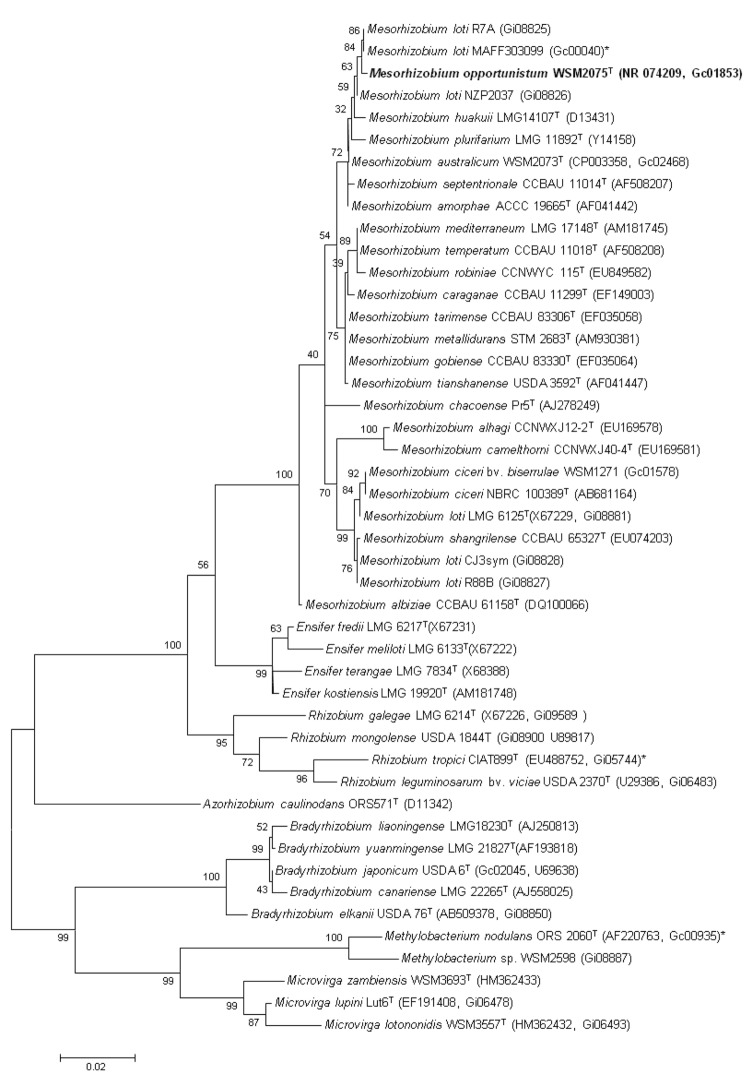
Phylogenetic tree showing the relationships of *Mesorhizobium opportunistum* WSM2075^T^ with other root nodule bacteria in the order *Rhizobiales* based on aligned sequences of the 16S rRNA gene (1,290 bp internal region). All positions containing gaps and missing data were eliminated. Phylogenetic analyses were performed using MEGA, version 3.1 [20]. The tree was built using the Maximum-Likelihood method with the General Time Reversible model and bootstrap analysis [21] with 500 replicates to construct a consensus tree. Type strains are indicated with a superscript T. Brackets after the strain name contain a DNA database accession number and/or a GOLD ID (beginning with the prefix G) for a sequencing project registered in GOLD [22]. Published genomes are indicated with an asterisk.

Figure 2 shows the phylogenetic neighborhood of *Mesorhizobium opportunistum* strain WSM2075^T^ in a 16S rRNA sequence based tree. This strain clusters in a tight group which included *M. amorphae, M. huakuii, M. plurifarium* and *M. septentrionale* and has >99% sequence identity with all four type strains. However, based on a polyphasic taxonomic study we have identified that this strain belongs to a new species [6].

### Symbiotaxonomy

*M. opportumistum* strain WSM2075^T^ forms an ineffective (non-N fixing) symbiosis with its original host of isolation, *B. pelecinus L.*, as well as with *Astragalus adsurgens*, *A. membranaceus, Lotus peregrinus* and *Macroptilium atropurpureum* [4,6]. In all cases the root nodules formed are small, white and seem incapable of fixing nitrogen [6]. Strain WSM2075^T^ has a broader host range for nodulation than *Mesorhizobium ciceri* bv. biserrulae WSM1271 [6].

## Genome sequencing and annotation

### Genome project history

This organism was selected for sequencing on the basis of its environmental and agricultural relevance to issues in global carbon cycling, alternative energy production, and biogeochemical importance, and is part of the Community Sequencing Program at the U.S. Department of Energy, Joint Genome Institute (JGI) for projects of relevance to agency missions. The genome project is deposited in the Genomes OnLine Database [22] and the complete genome sequence in GenBank. Sequencing, finishing and annotation were performed by the JGI. A summary of the project information is shown in Table 2.

**Table 2:** Genome sequencing project information for *Mesorhizobium opportunistum* WSM2075^T^.

**MIGS ID**	**Property**	**Term**
MIGS-31	Finishing quality	Finished
MIGS-28	Libraries used	Illumina GAii shotgun library, 454 Titanium standard library and paired end 454 libraries
MIGS-29	Sequencing platforms	Illumina and 454 technologies
MIGS-31.2	Sequencing coverage	454 std (63.8×), 454 paired end (91.5×) and Illumina (1×), total 146.9×
MIGS-30	Assemblers	Velvet, Newbler, phred/Phrap/Consed
MIGS-32	Gene calling method	Prodigal, GenePRIMP
	Genbank ID	CP002279
	Genbank Date of Release	January 21, 2011
	GOLD ID	Gc01853
	NCBI project ID	33861
	Database: IMG	2503198000
	Project relevance	Symbiotic nitrogen fixation, agriculture

### Growth conditions and DNA isolation

*M. opportunistum* strain WSM2075^T^ was grown to mid logarithmic phase in TY rich medium [23] on a gyratory shaker at 28°C. DNA was isolated from 60 mL of cells using a CTAB (Cetyl trimethyl ammonium bromide) bacterial genomic DNA isolation method [24].

### Genome sequencing and assembly

The genome of *Mesorhizobium opportunistum* WSM2075^T^ was sequenced at the Joint Genome Institute (JGI) using a combination of Illumina [25] and 454 technologies [26]. An Illumina GAii shotgun library comprising 370 Mb in reads of 36 bases, a 454 Titanium library with read length of 480-495 bases containing approximately 1.05 million reads, and a paired end 454 library containing 63840 reads with average insert size of 39 Kb were generated for this genome. All general aspects of library construction and sequencing performed at the JGI can be found at [24]. Illumina sequencing data was assembled with VELVET [27], and the consensus sequences were shredded into 1.5 Kb overlapped fake reads and assembled together with the 454 data. Draft assemblies were based on 375 Mb 454 standard data, and all of the 454 paired end data. Newbler parameters used were ‘-consed -a 50 -l 350 -g -mi 96 -ml 96’. The initial Newbler assembly contained 44 contigs in 1 scaffold. We converted the initial 454 assembly into a phrap assembly by making fake reads from the consensus, collecting the read pairs in the 454 paired end library. The Phred/Phrap/Consed software package was used for sequence assembly and quality assessment [28-30] in the subsequent finishing process. Illumina data was used to correct potential base errors and increase consensus quality using software developed at JGI (Polisher, Alla Lapidus, unpublished). After the shotgun stage, reads were assembled with parallel phrap (High Performance Software, LLC). Gaps were closed *in silico* using software developed at JGI (gapResolution, unpublished), and mis-assemblies were corrected using Dupfinisher [31], or sequencing cloned bridging PCR fragments. Remaining gaps between contigs were manually closed by editing in Consed, by PCR, and by Bubble PCR primer walks. A total of 464 additional reactions and 3 shatter libraries were necessary to close all gaps and to improve the quality of the finished sequence.

### Genome annotation

Genes were identified using Prodigal [32] as part of the Oak Ridge National Laboratory genome annotation pipeline, followed by a round of manual curation using the JGI GenePrimp pipeline [33]. The predicted CDSs were translated and used to search the National Center for Biotechnology Information (NCBI) nonredundant database, UniProt, TIGRFam, Pfam, PRIAM, KEGG, COG, and InterPro databases. These data sources were combined to assert a product description for each predicted protein. Non-coding genes and miscellaneous features were predicted using tRNAscan-SE [34], RNAMMer [35], Rfam [36], TMHMM [37], and SignalP [38]. Additional gene prediction analyses and functional annotation were performed within the Integrated Microbial Genomes (IMG-ER) platform [39].

## Genome properties

**Figure 3:**
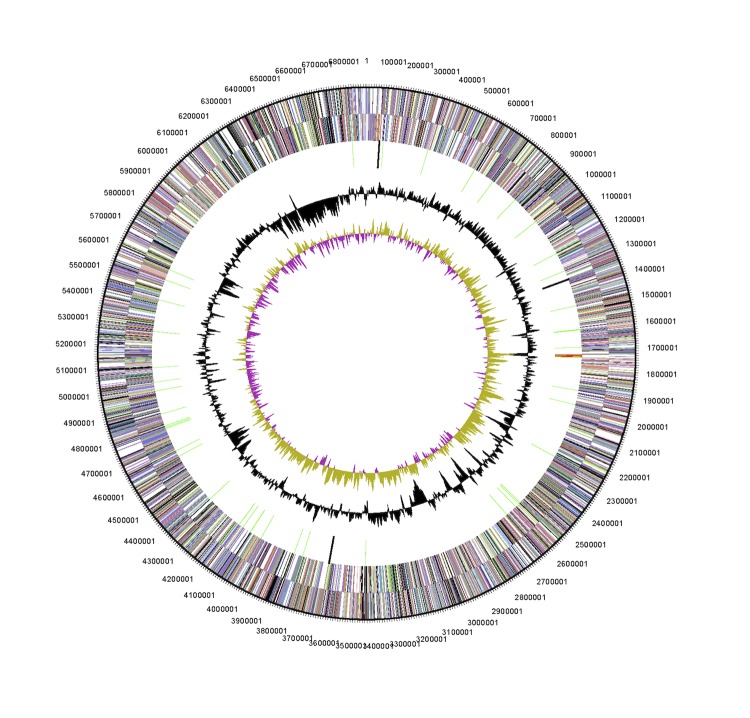
Graphical circular map of the chromosome of *Mesorhizobium opportunistum* WSM2075^T^. From outside to the center: Genes on forward strand (color by COG categories as denoted by the IMG platform), Genes on reverse strand (color by COG categories), RNA genes (tRNAs green, sRNAs red, other RNAs black), GC content, GC skew.

The genome is 6,884,444 nucleotides with 62.87% GC content (Table 3) and comprised of a single chromosome and no plasmids. From a total of 6,747 genes, 6,685 were protein encoding and 62 RNA only encoding genes. Within the genome, 177 pseudogenes were also identified. The majority of genes (71.11%) were assigned a putative function while the remaining genes were annotated as hypothetical. The distribution of genes into COGs functional categories is presented in Table 4 and Figure 3.

**Table 3:** Genome Statistics for *Mesorhizobium opportunistum* WSM2075^T^.

**Attribute**	**Value**	**% of Total**
Genome size (bp)	6,884,444	100.00
DNA coding region (bp)	5,948,427	86.40
DNA G+C content (bp)	4,328,075	62.87
Number of replicons	1	
Extrachromosomal elements	0	
Total genes	6,747	100.00
RNA genes	62	0.92
Protein-coding genes	6,685	99.08
Genes with function prediction	4,798	71.11
Genes assigned to COGs	5,353	79.34
Genes assigned Pfam domains	5,595	82.93
Genes with signal peptides	610	9.04
Genes with transmembrane helices	1,573	23.31
		

**Table 4:** Number of protein coding genes of *Mesorhizobium opportunistum* WSM2075^T^ associated with the general COG functional categories.

**Code**	**Value**	**%age**	**Description**
J	194	3.26	Translation, ribosomal structure and biogenesis
A	0	0	RNA processing and modification
K	515	8.65	Transcription
L	185	3.11	Replication, recombination and repair
B	5	0.08	Chromatin structure and dynamics
D	37	0.62	Cell cycle control, mitosis and meiosis
Y	0	0	Nuclear structure
V	63	1.06	Defense mechanisms
T	227	3.81	Signal transduction mechanisms
M	315	5.29	Cell wall/membrane biogenesis
N	50	0.84	Cell motility
Z	1	0.02	Cytoskeleton
W	1	0.02	Extracellular structures
U	131	2.2	Intracellular trafficking and secretion
O	208	3.5	Posttranslational modification, protein turnover, chaperones
C	353	5.93	Energy production conversion
G	592	9.95	Carbohydrate transport and metabolism
E	710	11.93	Amino acid transport metabolism
F	93	1.56	Nucleotide transport and metabolism
H	217	3.65	Coenzyme transport and metabolism
I	242	4.07	Lipid transport and metabolism
P	250	4.2	Inorganic ion transport and metabolism
Q	191	3.21	Secondary metabolite biosynthesis, transport and catabolism
R	777	13.06	General function prediction only
S	594	9.98	Function unknown
-	1394	20.66	Not in COGS
Total	5,951
